# A method for the production and expedient screening of CRISPR/Cas9-mediated non-transgenic mutant plants

**DOI:** 10.1038/s41438-018-0023-4

**Published:** 2018-03-02

**Authors:** Longzheng Chen, Wei Li, Lorenzo Katin-Grazzini, Jing Ding, Xianbin Gu, Yanjun Li, Tingting Gu, Ren Wang, Xinchun Lin, Ziniu Deng, Richard J. McAvoy, Frederick G. Gmitter, Zhanao Deng, Yunde Zhao, Yi Li

**Affiliations:** 1 Department of Plant Science and Landscape Architecture, University of Connecticut, Storrs, CT USA; 2 Institute of Vegetable Crops, Jiangsu Academy of Agricultural Sciences, Nanjing, China; 3 College of Horticulture and State Key Laboratory of Crop Genetics and Germplasm Enhancement, Nanjing Agricultural University, Nanjing, China; 4 State Key Laboratory of Subtropical Silviculture, Zhejiang Agriculture and Forestry University, Zhejiang Hangzhou, China; 5 College of Horticulture, Hunan Agricultural University, Hunan Changsha, China; 6 Citrus Research and Education Center, University of Florida, Lake Alfred, FL USA; 7 Department of Environmental Horticulture, Gulf Coast Research and Education Center, IFAS, University of Florida, Wimauma, FL USA; 8 Section of Cell and Developmental Biology, University of California at San Diego, San Diego, CA 92093 USA

## Abstract

Developing CRISPR/Cas9-mediated non-transgenic mutants in asexually propagated perennial crop plants is challenging but highly desirable. Here, we report a highly useful method using an *Agrobacterium*-mediated transient CRISPR/Cas9 gene expression system to create non-transgenic mutant plants without the need for sexual segregation. We have also developed a rapid, cost-effective, and high-throughput mutant screening protocol based on Illumina sequencing followed by high-resolution melting (HRM) analysis. Using tetraploid tobacco as a model species and the phytoene desaturase (*PDS*) gene as a target, we successfully created and expediently identified mutant plants, which were verified as tetra-allelic mutants. We produced *pds* mutant shoots at a rate of 47.5% from tobacco leaf explants, without the use of antibiotic selection. Among these *pds* plants, 17.2% were confirmed to be non-transgenic, for an overall non-transgenic mutation rate of 8.2%. Our method is reliable and effective in creating non-transgenic mutant plants without the need to segregate out transgenes through sexual reproduction. This method should be applicable to many economically important, heterozygous, perennial crop species that are more difficult to regenerate.

## Crop breeding: A new technique for using CRISPR/Cas9 to create non-transgenic mutant crop plants

A group led by Dr. Yi Li of University of Connecticut, USA has developed a technique to reliably create desirable mutations in crop plants without introducing any foreign DNA, thus generating non-transgenic mutant plants. Their technique will be particularly important for application of genome editing technologies in perennial crop plants. Genome editing technologies have been used to introduce desired mutations into plants but the approach normally incorporates foreign genes such as Cas9 into the plant’s genome. To avoid this, Dr. Li’s team has used Agrobacterium to transiently express the CRISPR/Cas9 components, a bacterium commonly used to genetically engineer plants. With their method, CRISPR/Cas9 from the bacteria edit the plant genome without introducing any foreign DNA, and then a two-step screening process is used to identify non-transgenic mutant plants. This new method provides a reliable and efficient alternative for producing non-transgenic genetically engineered crops.

## Introduction

Transgenic technologies provide powerful tools for crop improvement. However, the application of these technologies has been hampered by public apprehension toward potential food safety and gene flow concerns, resulting from the presence and/or expression of transgenes^1,2^. Recent development of CRISPR/Cas9-mediated genome editing has made targeted mutagenesis an attractive alternative to traditional transgenic technologies^3,4^. In plants, the most widespread application of CRISPR/Cas9 entails the stable integration of Cas9 endonuclease and single-guide RNA (sgRNA) genes into host genomes^5–16^. When CRISPR/Cas9-mediated gene editing is used in sexually propagated annual crop plants, transgenes (Cas9, sgRNA, and so on) can be eliminated from host genomes following sexual reproduction and screening of segregating populations. This segregation of CRISPR/Cas9 transgenes from mutations of interest can result in non-transgenic mutant plant progeny^17–20^. However, this strategy is rarely feasible or practical for vegetatively propagated perennial plants. These plants generally require years to reach sexual maturity; thus, multiple years are needed before sexual reproduction is feasible^21^. Additionally, these plants are highly heterozygous for genes controlling many important traits, and these traits will segregate and recombine following sexual reproduction, resulting in non-transgenic mutant progeny likely lacking a combination of desirable traits^22^.

Developing a method to generate CRISPR/Cas9-mediated non-transgenic mutants is highly desirable for many applications of genome editing, particularly for asexually propagated, heterozygous, perennial crop plants. It has been reported that pre-assembled CRISPR/Cas9 ribonucleoproteins can be delivered into protoplasts to induce mutations, without the need for stable integration of *CRISPR*/*Cas9* genes into the host-plant genome^23–25^. Particle bombardment has also been used to deliver CRISPR/Cas9 ribonucleoproteins to wheat and maize cells, producing non-transgenic mutants^26–29^. However, working with protoplasts, as well as utilizing biolistics, limits the potential for full-plant regeneration to some species and tissue types. Therefore, it is important to also develop alternative methods to produce non-transgenic CRISPR mutants of perennial crop plant species.

In contrast to the limited success of plant regeneration from protoplasts^30,31^, plant regeneration from leaf, hypocotyl, epicotyl, shoot, root, cotyledon, or callus explants has been well established for the majority of crop plant species^32^, including many that are recalcitrant to regeneration from protoplasts^33,34^. It has also been shown that proteins can be produced following transient expression of *Agrobacterium* T-DNA genes^35,36^. In addition, *Agrobacterium* inoculation protocols have been developed for many perennial crop species^37^. To circumvent the limited regeneration potential when using CRISPR ribonucleoproteins to produce non-transgenic mutants, we report a method for using *Agrobacterium* to transiently express the Cas9 and sgRNA genes in plant cells, using tobacco as a model plant and *PDS* as a model target gene. We have also developed a high-throughput screening protocol utilizing next-generation sequencing in combination with high-resolution DNA melting (HRM) analysis to efficiently identify mutants from a population of shoots regenerated in the absence of selection pressure. We demonstrate that the combination of *Agrobacterium*-mediated transient CRISPR/Cas9 expression with a highly efficient screening protocol makes it possible to efficiently obtain non-transgenic mutant plants, a method that should be applicable to heterozygous perennial crop species.

## Results

### Production of mutants via *Agrobacterium*-mediated expression without antibiotic selection

**Fig. 1:**
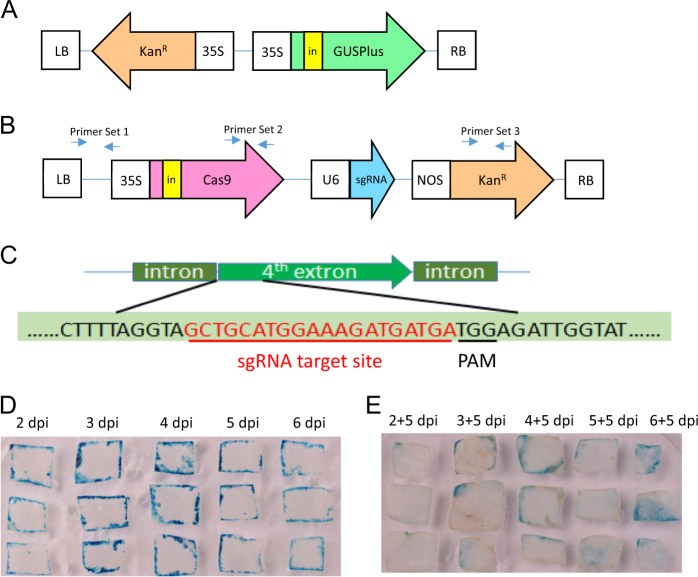
*Agrobacterium*-mediated transient expression of GUS and *CRISPR*/*Cas9* genes in tobacco. **a**, **b** T-DNA constructs for (**a**) GUS and (**b**) CRISPR/Cas9 (Cas9 and a *PDS*-targeted sgRNA) expression. The GUS construct consists of a GUSPlus gene interrupted by a cas1 intron (in), to prevent bacterial expression, under the control of a CaMV 35S promoter, and a kanamycin resistance gene (*Kan*^R^). **b** The CRISPR construct (hCas9-NtPDS) consists of a *Cas9* gene interrupted with an IV2 intron (in), an sgRNA targeting the tobacco *PDS* gene under the control of a U6 promoter, and a kanamycin (*Kan*^R^) resistance gene. Primer sets 1, 2, and 3 were used for PCR analysis to determine the presence of T-DNA fragments stably integrated into the tobacco genome. **c** The fourth exon of the tobacco *PDS* gene was selected as the target site for the sgRNA. **d**, **e** Histochemical staining of GUS activity in tobacco leaf discs (**d**) without or (**e**) with timentin to suppress *Agrobacterium* growth. **d** GUS activity in tobacco leaf discs 2–6 days following infection by *Agrobacterium*. **e** Reduction in GUS activity in tobacco explants transferred to timentin-containing media, to suppress *Agrobacterium* growth, for an additional 5 days following the initial 2–6 day co-incubation. The difference in GUS expression activities between explants in **d** and those in **e** indicated transient GUS expression demonstrating that transient GUS expression peaked at 3–4 days post infection

Using tobacco (*Nicotiana tabacum* Xanthi) as a model plant and an intron-containing *GUS* gene as a marker (Fig. 1a), we observed that transient expression of T-DNA genes in inoculated leaf discs peaked 3–4 days following *Agrobacterium* infection in the absence of kanamycin selection. Figure 1d shows the GUS activity in tobacco leaf discs 2–6 days post infection (dpi), and Fig. 1e shows the GUS activity in leaf discs after 5 additional days in timentin-containing media. The antibiotic timentin was used to suppress *Agrobacterium* growth following an initial 2–6 day co-incubation; thus, the GUS activities shown in Fig. 1e should result from stable integration of the *GUS* gene into the tobacco genome. The difference in GUS expression between explants in Fig. 1d and those in Fig. 1e is indicative of transient GUS expression, demonstrating that there are high levels of transient expression of the genes in the T-DNA region. The results in Fig. 1d, e indicated that a 3-day or 4-day co-incubation was optimal for *Agrobacterium-*mediated transient expression of the *GUS* gene. Three days of *Agrobacterium* co-incubation was subsequently used for transient expression of *CRISPR*/*Cas9* genes.

The sgRNA used in these experiments (Fig. 1b) targets the beginning of the fourth exon of the endogenous tobacco phytoene desaturase gene (*PDS*), as shown in Figure 1c. Previous studies have shown that disruption of this gene leads to an albino phenotype^38^. We used this phenotype as a visual marker to identify tobacco mutants whose *PDS* gene had been edited following the expression of Cas9 and *PDS*-targeting sgRNA genes. We infected 415 tobacco leaf-disc explants in three independent experiments using 3 days of *Agrobacterium* co-incubation without any selection for transgenic cells or shoots (Table 1). A total of 197 shoots regenerated from infected explants exhibited the albino phenotype, indicating a mutation in *PDS*, demonstrating a mutation rate of 0.475 *pds* mutants per explant. However, due to the lack of chemical selection, the total number of shoots regenerated from each explant was very high, and therefore, the mutation rate per total regenerated shoots was quite low (2.57%, Supplementary Table 1). These results indicate that *pds* mutant plants can be produced via *Agrobacterium*-mediated expression of *Cas9* and sgRNA genes without using antibiotic selection. Ten independent *pds* mutant plants were randomly chosen for further analysis, and six are shown in Table 2. The specific genetic mutations in these plant lines were identified via high-throughput sequencing, which demonstrated that all plants contained tetra-allelic mutations, meaning that all four alleles were mutagenized and no wild-type alleles could be detected. Microscopic analysis of plant tissues was unable to uncover the presence of green cells in albino *pds* mutants, suggesting that all *PDS* genes in all cells were mutated in these plants.

**Table 1:** *pds* mutant tobacco shoots regenerated without kanamycin selection

Transformation experiment	No. of explants^1^	Total no. of independent *pds* mutant shoots^2^	Mutation efficiency (%)^3^
1	159	53	33.3
2	88	59	67.1
3	168	85	50.6
Total	415	197	47.5

^1^The number of tobacco leaf explants infected with *Agrobacterium tumefaciens* harboring hCas9-NtPDS

^2^The total number of independent *pds* mutant shoots as indicated by an albino phenotype

^3^The mutation efficiency was calculated as follows: $(\frac {\mathrm {no}. \mathrm{of}\,pds\, \mathrm {mutant}\, \mathrm {shoots}} {\mathrm {no}. \mathrm {of}\, \mathrm {explants}}) \times 100$

**Table 2:** Mutations in individual mutant plant lines identified through high-throughput sequencing analyses

Mutant plant line	Sequence	Mutation description
Wild type	$\frac{A}{{43}}\frac{A}{{44}}\frac{A}{{45}}\frac{G}{{46}}\frac{A}{{47}}\frac{T}{{48}}\frac{G}{{49}}\frac{A}{{50}}\frac{T}{{51}}\frac{G}{{52}}\frac{A}{{53}}\frac{T}{{54}}\frac{G}{{55}}$	Not applicable
*pds-9*	AAAGATGA_GATG	1 bp deletion @ position 51
	AAAGATGATTGATG	1 bp insertion @ position 51 or 52 (T)
	AAAGAT_____TG	5 bp deletion @ positions 49–53
	AAAGATG__GATG	2 bp deletion @ positions 50 and 51
*pds-10*	AAAGAT__TGATG	2 bp deletion @ positions 49 and 50
	AAAGATGA__ATG	2 bp deletion @ positions 51 and 52
*pds-11*	AAAGAT__TGATG	2 bp deletion @ positions 49 and 50
	AAAGATGAGTGATG	1 bp insertion @ position 51 (G)
	AAAGATG__GATG	2 bp deletion @ positions 50 and 51
	AAAGATGA_____	48 bp deletion @ positions 51–98
*pds-12*	AAAGATGA_GATG	1 bp deletion @ position 51
	AA_____ATGATG	5 bp deletion @ positions 45–49
	AAAGAT__TGATG	2 bp deletion @ positions 49 and 50
	AAA____ATGATG	4 bp deletion @ positions 46–49
*pds-13*	AAAGAT_ATGATG	1 bp deletion @ position 49
	AAAGATG_TGATG	1 bp deletion @ position 50
	A______ATGATG	6 bp deletion @ positions 44–49
	AAAGATGAATGATG	1 bp insertion @ position 50 or 51 (A)
*pds-14*	AAAGATGATTGATG	1 bp insertion @ positions 51 or 52 (T)
	AAAGAT__TGATG	2 bp deletion @ positions 49 and 50
	AAA______GATG	6 bp deletion @ positions 46–51
	AAAGATGAATGATG	1 bp insertion @ position 50 or 51 (A)

The albinism resulting from the disrupted *PDS* gene enabled us to conveniently identify *pds* mutant shoots at early stages of shoot development in this study. However, the vast majority of desirable mutations for crop improvement are unlikely to display any visually identifiable phenotypes at the early stages of shoot development. When no selection pressure is applied during callus and shoot regeneration following *Agrobacterium* infection, the vast majority of regenerated shoots or plantlets should be non-mutant, as demonstrated above. Therefore, the ability to efficiently identify mutants lacking any visually identifiable phenotype from a population of regenerated shoots is essential for using the above-described *Agrobacterium*-mediated transient mutagenesis system. Toward this end, we tested the effectiveness and efficiency of a two-step screening method using the newly produced *pds* mutants. The first step, an initial identification of mutants, takes advantage of the high-throughput nature of Illumina sequencing, and the second step, a fine identification of mutants, makes use of the high resolution of HRM analysis.

### Initial screening of CRISPR-mediated mutants using high-throughput DNA sequencing analysis

Although our mutagenesis rate was relatively high per explant, without a visible phenotype, it would be difficult to identify mutant plants due to the high number of regenerated shoots in the absence of chemical selection. Additionally, other CRISPR mutagenesis projects could have an even lower mutation rate than the one reported here. Therefore, we have developed a two-step method for high-throughput screening of shoots to identify the presence of targeted mutations. We first mixed leaf tissue from an albino *pds* mutant (MT), *pds-12*, with leaf tissues from independently derived non-mutant shoots (WT), regenerated from *Agrobacterium-*infected explants, at MT-to-WT ratios of 1:20, 1:41, and 1:83. We isolated genomic DNA from these pooled tissue samples and performed PCR reactions to amplify a 186-bp fragment that contained the sgRNA-target region on the fourth *PDS* exon (Fig. 1c). PCR products were sequenced on an Illumina platform to ~×60,000 to ×100,000 coverage. Next, we measured the amount of PCR product derived from the MT-to-WT ratios of 1:20, 1:41, and 1:83 mixed tissues and diluted it with ×6 the amount of PCR product (ng) derived from WT plant tissue. The diluted PCR product was also used for Illumina sequencing analysis.

**Fig. 2:**
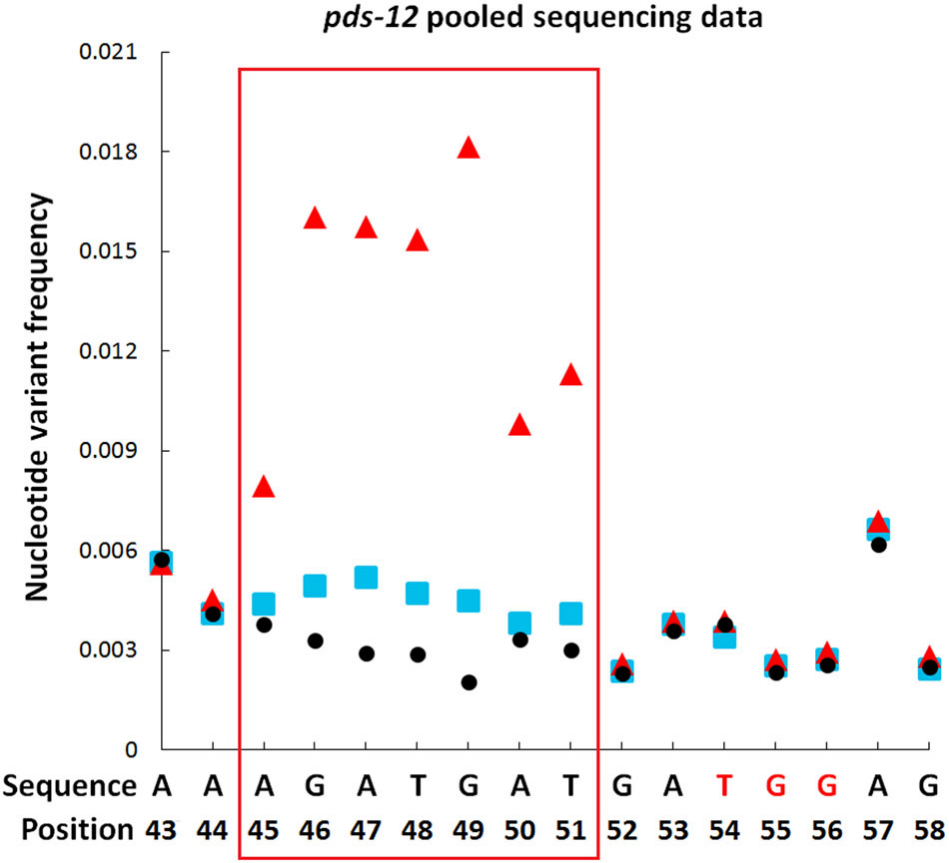
CRISPR/Cas9-mediated mutations were identified with high-throughput sequencing analysis of the PCR-amplified sgRNA-target region of the *pds-12* mutant. The red-marked TGG sequence on the *x* axis represents the three PAM nucleotides. Black circles represent nucleotide variant frequencies (NVF) of PCR product amplified from wild-type (WT) DNA. Red triangles represent the NVF of PCR product amplified from the genomic DNA of a 42-plant pool containing the *pds-12* mutant line, displaying much higher NVF at positions 45–51. Blue squares represent the NVF of the same PCR products as the red triangles, but diluted six times with the WT PCR product, demonstrating that the NVF at nucleotide positions 45–51 are significantly reduced following the dilution. Using the WT PCR product dilution method, mutations at positions 45–51 were identified, consistent with the DNA sequencing results of the *pds-12* mutant plant (Table 2)

We observed that the PCR products derived from a 42-plant (1MT: 41WT) pooled tissue sample containing the *pds-12* mutant showed a drastically elevated nucleotide variant frequency (NVF) at positions 45–51 (red triangles, Fig. 2, showing a 1MT: 41WT pooled tissue sample), which is consistent with verified mutations at these nucleotide positions (Table 2). NVF is a measure of the frequency of abnormal (compared to WT reference) nucleotides detected by DNA sequencing at a given position due to mutations or sequencing error. When we diluted the same PCR products with 6× WT PCR products (blue squares, Fig. 2), we observed significant reductions in NVF at positions 45–51 relative to the undiluted PCR product. The observed elevations and reductions of NVF before and after a 6× WT DNA dilution further verified the presence of mutations at nucleotide positions 45–51, as NVF resulting from sequencing error would be unaffected by dilution. As shown in Table 2, high-throughput sequencing uncovered four types of mutations in the *pds-12* mutant line: a 1-bp deletion at position 51, a 5-bp deletion at positions 45–49, a 2-bp deletion at positions 49–50, and a 4-bp deletion at positions 46–49 (Table 2). Similar results were observed using the 1MT: 20WT and 1MT: 83WT pooled tissue samples, with a more drastic elevation of NVF for the 1MT: 20WT samples and reduced elevated NVF for the 1MT: 83WT samples compared to the 1MT: 41WT samples (data not shown).

Using a single-blind approach (the researchers who conducted the experiment did not know which 42-plant groups contained *pds* mutants), we tested the accuracy of the mutant screening method based on elevations and reductions of NVF before and after a 6× WT DNA dilution as described above. We created eight 42-plant pools, five of which contained plant tissue from a single *pds* mutant plant line and three of which contained 100% WT plants. The five *pds* mutants used were *pds-9*,* pds-10*,* pds-11*, *pds-13*, and *pds-14*. We confirmed that the screening method was reliable for identifying all 42-plant pools that contained *pds* mutant plants at a ratio of 1MT: 41WT (Supplementary Figure 1), with 100% accuracy (Table 3). Thus, the elevations and reductions of NVF before and after a 6× WT DNA dilution were excellent indicators of 42-plant pools that contained mutant plants.

**Table 3:** Accuracy of mutation identification via high-throughput sequencing analysis based on pooled samples of 42 plants

Mutant	Mutant loci identified via DNA sequencing for pooled samples of 42 plants^1^	Mutation verification by sequencing the target DNA region of individual mutant plant lines^2^	Accuracy
*pds-9*	Nucleotide positions 49–53	Deletions at nucleotide positions 49–53, insertion at positions 51 or 52	100%
*pds-10*	Nucleotide positions 49–52	Deletions at nucleotide positions 49–52	100%
*pds-11*	Nucleotide positions 49–98	Deletions at nucleotide positions 49–98, and an insertion at position 51	100%
*pds-12*	Nucleotide positions 45–51	Deletions at nucleotide positions 45–51	100%
*pds-13*	Nucleotide positions 44–50	Deletions at nucleotide positions 44–50, and insertions at positions 50 or 51	100%
*pds-14*	Nucleotide positions 46–51	Deletions at nucleotide positions 46–51, insertions at positions 50 or 51 and 51 or 52	100%
WT	None detected	None	100%

^1^Mutant loci identified by a high-throughput sequencing method described in Fig. 2 for six randomly selected mutant plant lines where deletions, insertions, or substitutions were involved. See supplementary Figure 1 for detailed data

^2^Verification of mutations was done by sequencing PCR products of individual mutant plant lines (shown in Table 2)

### Fine identification of mutants using DNA high-resolution melting analysis

**Fig. 3:**
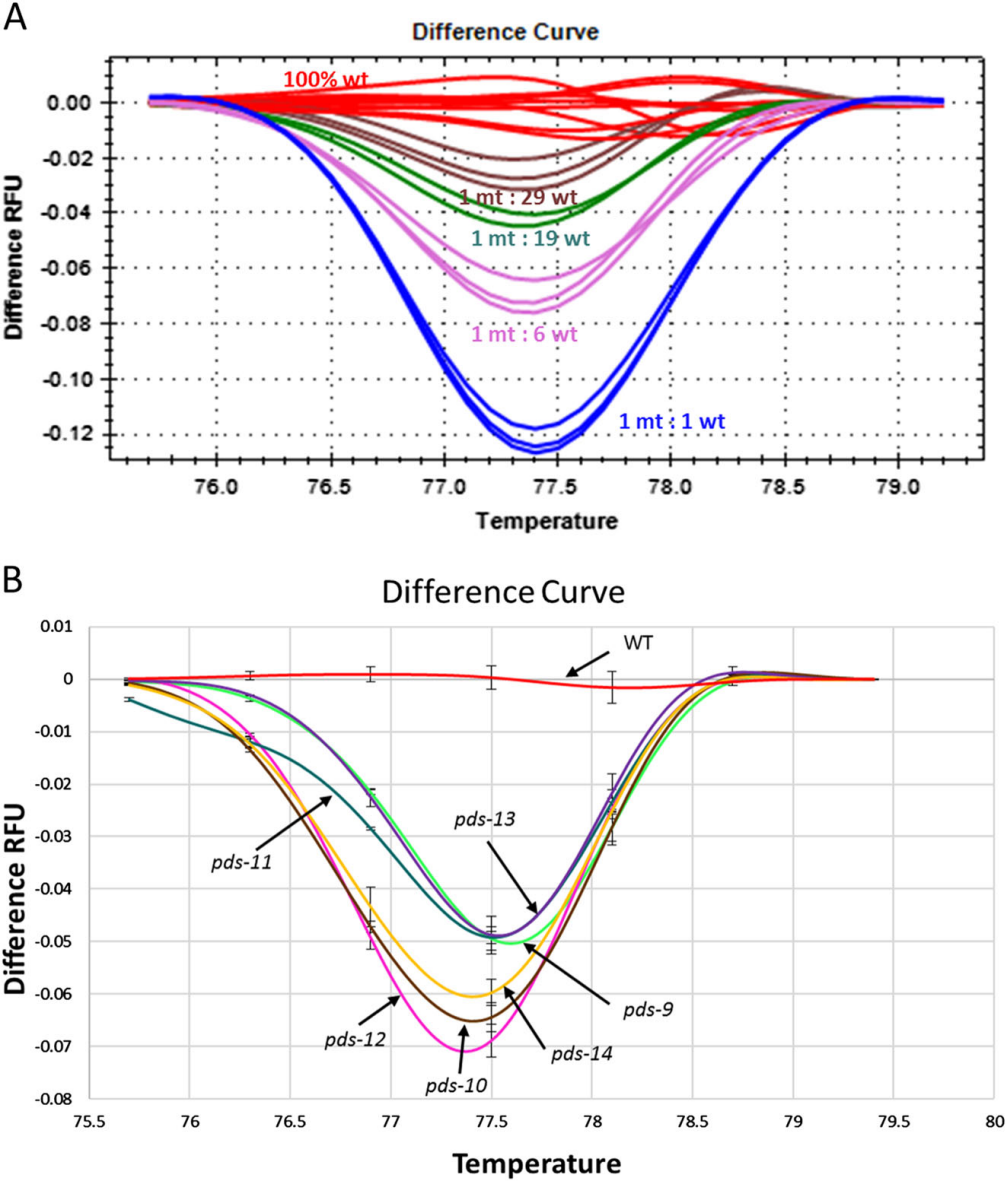
Identification of mutant plants using high-resolution melt (HRM) analysis of PCR products derived from pooled plant samples. **a** HRM analysis of DNA from pooled samples containing *pds-12* with various ratios of mutant (MT) to wild-type (WT) plant tissue. The pooled samples were as follows: a 100% WT plant pool, a 2-plant pool (1MT + 1WT), a 7-plant pool (1MT + 6WT), a 20-plant pool (1MT + 19WT), and a 30-plant pool (1MT + 29WT). Melt curves demonstrate that the 20-plant pool sample can be clearly distinguished from those of the 100% WT pool. **b** HRM analysis of the 7-plant pool containing different *pds* mutants. HRM curves of the 100% WT pool (negative control) or 7-plant pools containing a single *pds-9*, *pds-10*, * pds-11*, *pds-12*, *pds-13*, or *pds-14* mutant demonstrate that HRM analysis is effective to identify single mutants from pooled samples of 7-plants. The WT curve is representative of the three negative control pools used in this single-blind screen. Difference RFU values in **b** represent the average of three replicates at each temperature point. Error bars show variance for each temperature point for each mutant line

After identification of mutant-containing 42-plant pools, HRM analysis was used to identify individual mutant plant lines within each of the 42-plant pools (Supplementary Figure 2). To determine the sensitivity of HRM analysis, we performed HRM analysis on PCR products amplified from various DNA templates combined at different ratios. These template mixes were created using one-part *pds-12* plant tissue combined with different parts independently regenerated non-mutant (WT) tissues in the following ratios: 1:1, 1:6, 1:19, and 1:29. Figure 3a shows that mutant-containing PCR products at ratios of 1:1, 1:6, and 1:19 (MT:WT) could be distinguished from a wild-type DNA reference. The plant pool size we chose for subsequent HRM analysis was 7; thus, each 42-plant pool containing DNA from mutants could be divided into six pools of seven plants each.

We also used a single-blind experiment approach to test the accuracy of HRM analysis to identify mutant plants. We created eight additional 7-plant pools, five containing a single *pds* mutant each (*pds-9*, *pds-10*, *pds-11*,* pds-13*, or *pds-14*), and the remaining three negative control pools containing only wild-type plants. The HRM analysis results are shown in Fig. 3b and demonstrate that all pooled samples containing *pds* mutant plants could be identified with 100% accuracy. Finally, upon the identification of mutant-containing 7-plant pools, individual mutant(s) within each pool were identified via HRM based on a 1:1 mix between each putative mutant and a WT plant. Through this method, we successfully identified all *pds* mutant plants (Table 2).

### Determination of non-transgenic *pds* mutants

**Fig. 4:**
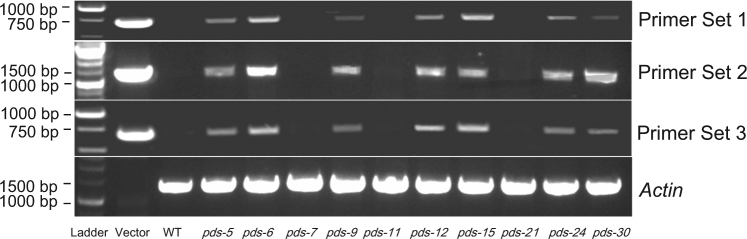
PCR verification of non-transgenic *pds* mutant lines using three primer sets (Fig. 1b) targeted to the three different regions of the T-DNA insert. Primer set 1 was used to amplify the T-DNA region between the left T-DNA border and the 5′ end of the CaMV 35S promoter. Primer set 2 was used to amplify the 3′ end of the Cas9 coding sequence. Primer set 3 was used to amplify a region of the kanamycin resistance gene (*Kan*^R^). The absence of all three T-DNA PCR fragments was indicative of non-transgenic plants. WT wild type

To distinguish transgenic from non-transgenic mutants, we performed PCR on 29 randomly selected *pds* mutant lines using primers targeted to three regions in the T-DNA fragment (Fig. 1b). Mutant plants were considered to be non-transgenic if they lacked a PCR product for all three primer sets (Fig. 4). Approximately 17.2% of the tested *pds* mutant lines were determined to be non-transgenic following PCR analysis (Table 4). As shown in Supplementary Figure 3, a non-transgenic plant (*pds-7*), along with a transgenic plant (*pds-9*), was cultured on MS media containing 100 mg/L kanamycin. The non-transgenic *pds-7* plant died under kanamycin selection, while the transgenic *pds-9* plant grew normally.

**Table 4:** Rate of non-transgenic *pds* mutants among all *pds* mutants

Transformation experiment	No. of independent *pds* mutant shoots characterized^1^	No. of non-transgenic *pds* mutant shoots identified^2^	% of characterized mutant shoots that were non-transgenic^3^
1	10	2	17.2
2	10	1	
3	9	2	

^1^A total of 29 *pds* mutant plants were chosen for identification of non-transgenic mutants

^2^The absence of the transgenes in *pds* mutant plants was verified by PCR and a kanamycin resistance test

^3^Percentage was calculated by dividing the total number of non-transgenic mutants by the total number of mutant plants analyzed and multiplying by 100

## Discussion

Producing non-transgenic mutants of heterozygous perennial crop plants using CRISPR/Cas9 technology is highly desirable but challenging. We developed an effective method for producing and identifying CRISPR/Cas9-mediated non-transgenic mutant plants, which should be applicable to many perennial heterozygous crop plants. We have demonstrated that we can use *Agrobacterium* to transiently express *CRISPR*/*Cas9* genes, and such expression can lead to the production of tetra-allelic, non-transgenic mutant plants. We have also demonstrated that the first step of our mutant identification, based on elevated and reduced nucleotide variance frequencies before and after a WT DNA dilution, using high-throughput DNA sequencing analysis, is reliable and highly efficient. Furthermore, the second step of mutant identification, using HRM analysis, is simple and effective.

With one sgRNA targeting the tobacco *PDS* gene, we achieved a 47.5% mutation rate (i.e., 0.475 *pds* mutants per explant) using no selective pressure during callus or shoot regeneration. At least 17.2% of the *pds* mutant plants produced this way were non-transgenic, for an overall non-transgenic mutation rate of 8.2% (i.e., 0.082 *pds* mutants per explant). We expect that the rate of recovery for non-transgenic mutant plants following *Agrobacterium*-mediated transient expression of *CRISPR*/*Cas9* genes could be much higher than we reported here. One reason is that the albino phenotype caused by *pds* mutations used in this study can result in cell- or shoot-growth disadvantages, which may have contributed to lower rates of mutant shoot production. Additionally, multiple sgRNA sequences may be used to target the same gene^39^ to increase the efficiency of mutant production.

Protoplast-mediated delivery of CRISPR/Cas9 ribonucleoproteins offers advantages for creating non-transgenic mutant plants^23–28,40^. However, regenerating plants from protoplasts can be difficult and has not been demonstrated to be possible for many important crop species. Low efficiency of plant regeneration from protoplasts has been reported in economically important crops such as avocado (*Persea americana*)^41^, grape (*Vitis vinifera*)^42^, and apple (*Malus domestica*)^43^. Furthermore, regeneration protocols have not been successfully demonstrated in many other plant species^31,44^. In the case of citrus, for instance, a globally important fruit crop that is often heterozygous for important trait genes, protoplast regeneration can be achieved only if protoplasts are derived from juvenile tissues^45^. However, plants generated from juvenile citrus tissues require years to reach a mature fruit production stage, and this delay is an impediment to the efficient evaluation of gene functions in fruit and for other genes pertinent to citrus breeding programs^46,47^. On the other hand, tissues from mature citrus trees, such as shoot segments, can be transformed with *Agrobacterium*^48^.

Jacobs et al.^49^ discussed the possibility of using *Agrobacterium*-mediated transient expression of *Cas9* and sgRNA genes to produce non-transgenic mutant plants. Iaffaldano et al.^50^ reported the production of CRISPR/Cas9-mediated mutant plants using *Agrobacterium* without any selection of stable transgenic plants. However, Iaffaldano et al.^50^ did not characterize whether their mutant plants were transgenic or non-transgenic, nor did they calculate the percentage of each type. The non-transgenic mutation rate reported in this study is lower than mutation rates for the previously reported protoplast microinjection methods^25^ but higher than for particle bombardment methods (per explant)^26–28^. However, *Agrobacterium* can be a more versatile tool because of its ability to efficiently infect many different tissues types across most plant species^51,52^, and it is the most widely used method for plant transformation. Thus, protocols for *Agrobacterium* infection and subsequent callus and shoot regeneration are readily available for many crop plant species. Furthermore, even when other methods are applicable, *Agrobacterium* infection procedures are relatively simple and easy to perform for many plant species. Thus, the method presented here provides an alternative method for the production of non-transgenic mutant plants.

Our method of using *Agrobacterium* to transiently express *CRISPR*/*Cas9* genes, without the use of chemical selection such as kanamycin, also offers some other advantages. The lack of a chemical selection agent following *Agrobacterium* infection allows for even greater rates of plant regeneration compared to when chemical selection is used and therefore likely enhances mutant callus/shoot production. This is because selection agents, such as kanamycin, hygromycin, and various herbicides, can suppress shoot regeneration for many plant species, even when those shoots express relevant resistance genes^53^. Furthermore, our high-throughput screening method, based on a novel DNA sequencing strategy in combination with HRM analysis, makes mutant identification fast and easy to perform. The drawback of our method is that there is a risk of creating transgenic mutant plants; however, identifying and discarding these plants does not add any significant time to the screening process.

We have observed that the threshold of mutant plant detection using the Illumina sequencing method can be much more sensitive than the detection of one mutant out of 42 plants (the 1MT: 41WT) described in this study. The use of the 42-plant sample pools, therefore, assures that the presence of a mutant plant can be readily detected in pooled samples. Additionally, the threshold of HRM analysis to detect mutant plants can be as low as 1 of 19 plants, but we chose one out seven (1MT: 6WT). Again, this pooling choice provides a safe margin for error by reducing the chance of missing mutant plants due to false negatives. Furthermore, a small number of false positives from the sequencing analysis of 42-plant pools are not a concern because they can be easily identified in the subsequent step of screening. Thus, our method, which combines a unique DNA sequencing strategy with HRM analysis, provides a reliable protocol to screen for CRISPR/Cas9-mediated mutant shoots from a population of shoots regenerated in the absence of selection pressure. The overall protocol for mutant identification is schematically represented in Supplementary Figure 4. Using this protocol, it would take ~14 days to identify all mutants from 1000 independent shoots that were subjected to *Agrobacterium*-mediated transient expression of *CRISPR*/*Cas9* genes.

In conclusion, we have developed a highly useful method utilizing *Agrobacterium-*mediated transient expression of *Cas9* and sgRNA genes combined with multi-step pooled screening, enabling the reliable production and efficient identification of non-transgenic mutants regenerated in the absence of selection pressure. Due to the versatility of CRISPR/Cas9 and *Agrobacterium*-mediated infection, as well as the ease of plant regeneration from leaf, shoot, or root explants, this method is applicable to many economically important plant species, particularly heterozygous perennial plant species that are recalcitrant to regeneration from protoplasts or following biolistic bombardment.

## Materials and methods

### Gene constructs

To determine the optimum experimental conditions for maximum transient expression of T-DNA in plant cells, we used pCAMBIA1305.1, a plasmid containing an intron-containing *GUS* gene under the control of a CaMV 35S promoter and a NOS terminator. We modified this construct by adding a kanamycin resistance gene (*NPTII*) under the control of a CaMV 35S promoter and CaMV 35S terminator using the KpnI and XbaI restriction enzymes (Fig. 1a). For CRISPR/Cas9-based disruption of the tobacco *PDS* gene, we made a number of modifications to the pK7WGF2::hCas9 plasmid (Addgene plasmid #46965). First, we added an IV2 intron to a *Cas9* gene to prevent Cas9 expression in *Agrobacterium*, this gene was used to replace the original Cas9 DNA in the vector using the SpeI and ApaI restriction enzyme sites. Second, we added an sgRNA targeting the fourth exon of the *PDS* gene in *Nicotiana benthamiana*^54^ under the control of the *Arabidopsis thaliana* U6–26 gene promoter (accession # At3G13855), using the XbaI and KpnI restriction enzymes. All of the added polynucleotides were synthesized by Genscript Corporation (Piscataway, NJ, USA). The final CRISPR/Cas9-containing plasmid was named hCas9-NtPDS (Fig. 1b).

### *Agrobacterium*-mediated transformation for transient expression

*A. tumefaciens* (EHA105) was used to deliver Ti-plasmid DNA into tobacco leaf-disc explants. *Agrobacterium* cells were cultured overnight (16 h, 200 r.p.m., 28 °C) in 5 mL of liquid LB medium containing 100 mg/L spectinomycin. The overnight culture was diluted with 50 mL of fresh liquid LB media (1:10 dilution) and then grown for 6–8 h (200 r.p.m., 28 °C). *Agrobacterium* cells were collected at an OD_600_ of 0.6–0.8, centrifuged at 5000 r.p.m. for 15 min, and finally re-suspended in 50 mL of liquid MS medium containing 100 µM acetosyringone (AS). Re-suspended bacterial cells were shaken (180 r.p.m., 28 °C) for 1 h before use. Tobacco leaf discs (0.5 cm^2^, from vegetatively propagated clonal plants) were incubated in *Agrobacterium* cell solution for 20 min. Explants were blotted dry on sterile filter paper and transferred onto solid MS media containing 100 µM AS and no antibiotics.

### Histochemical GUS activity assay

To optimize the time needed for robust transient T-DNA expression, tobacco leaf explants inoculated with *Agrobacterium* (containing the pCAMBIA1305.1 vector) were incubated in the dark at 25 °C for 2, 3, 4, 5, or 6 days. Half of the leaf discs (six) from each treatment were stained for GUS activity^55^. The remaining explants were transferred to solid MS medium containing 150 mg/L timentin to suppress *Agrobacterium* growth. These explants were stored in the dark for an additional 5 days. Leaf discs from each treatment were histochemically stained to estimate stable versus transient expression levels of the *GUS* gene.

### Callus and shoot regeneration

For transient expression of the *CRISPR*/*Cas9* genes, tobacco leaf discs were incubated in the dark at 25 °C for 3 days before being transferred to solid media containing MS salts, 2.0 mg/L benzylaminopurine (to promote shoot initiation), and 150 mg/L timentin (to repress *Agrobacterium* growth). No antibiotics for the selection of transgenic plant cells were included in the culture media. The plates were stored at 25 °C under a 16-h photoperiod. After 4 weeks, visual screening was performed to identify white-colored mutant shoots. The white shoots were then transferred to solid MS media containing 150 mg/L timentin for rooting. Genomic DNA was isolated using the NucleoSpin plant II kit (Macherey-Nagel, Dueren, Germany). The primer pair PDS-F (5′-CTGAAGCAGTCACCAAGA-3′) and PDS-R (5′-AGTACGCATTCTTGAGGAGTC-3′) was used to amplify the sgRNA-target region. A second round of PCR was performed, with the primer pair PDS-FA (5′–**ACACTCTTTCCCTACACGACGCTCTTCCGATCT**CTGAAGCAGTCACCAAGA-3′) and PDS-RA (5′–**GTGACTGGAGTTCAGACGTGTGCTCTTCCGATCT**AGTACGCATTCTTGAGGAGTC-3′), using the PCR product of the first round as the template to add general adapter sequences to the PCR products. A final round of PCR was performed to add a barcode tag to the PCR products, with the primer pair PDS-FAI (5′-**AATGATACGGCGACCACCGAGATCTACAC-index-**ACACTCTTTCCCTACACGA-3′) and PDS-RAI (**5**′**-CAAGCAGAAGACGGCATACGAGAT-index-**GTGACTGGAGTTCAGACGTG-3′). Sequencing reactions were performed using the barcoded PCR products and the Illumina MiSeq platform (paired-end ×2 150 bp read length). The raw Illumina reads were mapped to the 186-bp reference sequence in the tobacco genome using bwa mem (-c 300000 –v 2) of BWA v0.5.9^56^ to determine the nature of mutations.

### High-throughput sequencing

For the 21-, 42-, and 84-plant pools used in the high-throughput sequencing screen, leaf tissue from *pds-12* mutant plants was combined with an equal amount of tissue from each of 20, 41, or 83 independently regenerated non-mutant shoots, respectively. One control pool was created using the tissue from 84 independently regenerated non-mutant shoots. Genomic DNA was isolated, amplified, tagged, and barcoded as described previously. For dilution, the PCR product amplified from the 42-plant pool was quantified using a NanoDrop and was then diluted with ×6 the amount of PCR product (ng) amplified from a pool composed of 84 independently regenerated non-mutant shoots. Sequencing reactions were performed in triplicate using the barcoded PCR products and the Illumina MiSeq platform (paired-end ×2 150 bp read length). The raw Illumina reads were mapped to the 186-bp reference sequence in the tobacco genome using bwa mem (-c 300000 –v 2) of BWA v0.5.9^56^. NVFs were calculated over a 77-bp region. To confirm the high-throughput screening method, five additional mutant-containing 42-plant pools were created, each comprising tissue from a single mutant plant (*pds-9*, *pds-10*, *pds-11*, *pds-13*, or *pds-14*) in combination with tissue from 41 independently regenerated non-mutant shoots. Three WT shoot control pools were created using the tissue from 42 independently regenerated non-mutant shoots. All eight pools were given randomly generated labels to assure that high-throughput sequencing screening could be performed in a single-blind manner.

### HRM analysis

For 2-, 7-, 20-, and 30-plant pools used in the high-resolution melting analysis screen, leaf tissue from *pds-12* mutant plants was combined with an equal amount of tissue from each of 1, 6, 19, or 29 independently regenerated non-mutant shoots, respectively. One control pool was created using the tissue from 30 independently regenerated non-mutant shoots. Genomic DNA was isolated using the NucleoSpin plant II kit (Macherey-Nagel). We used primers PDS-F and PDS-R (described previously) to amplify the sgRNA-target region. We also tested 85 bp (F primer: 5′-ATCTGTTCTGCACCTGAATAC-3′, R primer: 5′-AACCCAGTCTCATACCAA-3′) and 146 bp (F primer: 5′-CTGAAGCAGTCACCAAGA-3′, R primer: 5′-AACCCAGTCTCATACCAA-3′) PCR products for HRM analysis. The 186-bp fragments were used for our experiments, as shown in our results. PCR amplifications were performed in 10 µL volumes containing 5 µL of Precision Melt Supermix (Bio-Rad, USA), 0.5 µL of each 2 µM primer, and 50 ng of genomic DNA. PCR was carried out in triplicate using 96-well white-walled PCR plates and a CFX96™ Real-Time PCR Detection System (Bio-Rad). The amplification started with an initial denaturation step at 95 °C for 5 min, followed by 50 cycles of 95 °C for 10 s, 60 °C for 30 s, and 72 °C extension for 1 min. High-resolution melting analyses of the PCR amplicons were carried out in the same plate immediately following PCR amplification, using the following cycle: 95 °C for 30 s and 60 °C for 1 min. Melting curves were generated over a 65–95 °C range with 0.2 °C increment. Melting curves were analyzed using Precision Melt Analysis software (Bio-Rad). To confirm our HRM screening method, five mutant-containing 7-plant pools were created, each comprised tissue from a single mutant plant (*pds-9*,* pds-10*,* pds-11*, *pds-13*, or *pds-14*) in combination with tissue from six independently regenerated non-mutant shoots. Three control pools were created using the tissue from seven independently regenerated non-mutant shoots. The eight pools were labeled randomly to assure that HRM screening was performed in a single-blind manner.

### Determination of non-transgenic mutant plants

Stable integration of the CRISPR/Cas9 T-DNA was determined by PCR analysis of the tobacco genomic DNA. Primers 1F (5′-AGGTGGCGAAGTCATCTGC-3′) and 1R (5′-TGTCGTTTCCCGCCTTCAG-3′) were used to amplify a 701-bp region of the T-DNA fragment. Primers 2F (5′-GCCTGTTTGGTAATCTTATCGC-3′) and 2R (5′-TCTTTCCACTCTGCTTGTCTCG-3′) were used to amplify a 1326-bp fragment of the *Cas9* gene. Primers 3F (5′-ACTGGGCACAACAGACAATC-3′) and 3R (5′-ACCGTAAAGCACGAGGAA-3′) were used to amplify a 668-bp fragment of the kanamycin resistance gene. The absence of PCR products from all three primer sets was deemed to be indicative of a lack of stable transgene integration into the tobacco genome, thus indicating a non-transgenic mutant plant. Shoots from *pds* mutant plants were cultured on MS media containing 100 mg/L kanamycin and allowed to grow for 35 days, producing further evidence for the lack of transgene insertion into the genome.

## Electronic supplementary material

Supplemental Materials

## References

[1] Li W (2017). Elevated auxin and reduced cytokinin contents in rootstocks improve their performance and grafting success. Plant Biotechnol. J..

[2] Tuteja N, Verma S, Sahoo RK, Raveendar S, Reddy IB (2012). Recent advances in development of marker-free transgenic plants: regulation and biosafety concern. J. Biosci..

[3] Cong L (2013). Multiplex genome engineering using CRISPR/Cas systems. Science.

[4] Mali P (2013). RNA-guided human genome engineering via Cas9. Science.

[5] Bortesi L, Fischer R (2015). The CRISPR/Cas9 system for plant genome editing and beyond. Biotechnol. Adv..

[6] Čermák T (2017). A multipurpose toolkit to enable advanced genome engineering in plants. Plant Cell.

[7] Feng Z (2013). Efficient genome editing in plants using a CRISPR/Cas system. Cell Res..

[8] Gil-Humanes J (2017). High‐efficiency gene targeting in hexaploid wheat using DNA replicons and CRISPR/Cas9. Plant J..

[9] Lawrenson T (2015). Induction of targeted, heritable mutations in barley and *Brassica oleracea* using RNA-guided Cas9 nuclease. Genome Biol..

[10] Lu Y, Zhu JK (2017). Precise editing of a target base in the rice genome using a modified CRISPR/Cas9 system. Mol. Plant.

[11] Mao Y (2013). Application of the CRISPR–Cas system for efficient genome engineering in plants. Mol. Plant.

[12] Xie K, Yang Y (2013). RNA-guided genome editing in plants using a CRISPR–Cas system. Mol. Plant..

[13] Xiong JS, Ding J, Li Y (2015). Genome-editing technologies and their potential application in horticultural crop breeding. Hortic. Res..

[14] Gao Y (2015). Auxin binding protein 1 (ABP1) is not required for either auxin signaling or *Arabidopsis* development. Proc. Natl Acad. Sci. USA.

[15] Gao Y, Zhao Y (2014). Specific and heritable gene editing in *Arabidopsis*. Proc. Natl Acad. Sci. USA.

[16] Ma X (2015). A robust CRISPR/Cas9 system for convenient, high-efficiency multiplex genome editing in monocot and dicot plants. Mol. Plant.

[17] Char SN (2017). An *Agrobacterium*‐delivered CRISPR/Cas9 system for high‐frequency targeted mutagenesis in maize. Plant Biotechnol. J..

[18] Zhang H (2014). The CRISPR/Cas9 system produces specific and homozygous targeted gene editing in rice in one generation. Plant Biotechnol. J..

[19] Zhou H, Liu B, Weeks DP, Spalding MH, Yang B (2014). Large chromosomal deletions and heritable small genetic changes induced by CRISPR/Cas9 in rice. Nucleic Acids Res..

[20] Gao X, Chen J, Dai X, Zhang D, Zhao Y (2016). An effective strategy for reliably isolating heritable and Cas9-free *Arabidopsis* mutants generated by CRISPR/Cas9-mediated genome editing. Plant Physiol..

[21] Artlip TS, Wisniewski ME, Arora R, Norelli JL (2016). An apple rootstock overexpressing a peach CBF gene alters growth and flowering in the scion but does not impact cold hardiness or dormancy. Hortic. Res..

[22] Norelli JL (2017). Genotyping-by-sequencing markers facilitate the identification of quantitative trait loci controlling resistance to *Penicillium expansum* in *Malus sieversii*. PLoS ONE.

[23] Malnoy M (2016). DNA-free genetically edited grapevine and apple protoplast using CRISPR/Cas9 ribonucleoproteins. Front. Plant Sci..

[24] Subburaj S (2016). Site-directed mutagenesis in *Petunia*×hybrida protoplast system using direct delivery of purified recombinant Cas9 ribonucleoproteins. Plant Cell Rep..

[25] Woo JW (2015). DNA-free genome editing in plants with preassembled CRISPR-Cas9 ribonucleoproteins. Nat. Biotechnol..

[26] Liang Z (2017). Efficient DNA-free genome editing of bread wheat using CRISPR/Cas9 ribonucleoprotein complexes. Nat. Commun..

[27] Svitashev S, Schwartz C, Lenderts B, Young JK, Cigan AM (2016). Genome editing in maize directed by CRISPR–Cas9 ribonucleoprotein complexes. Nat. Commun..

[28] Zhang Y (2016). Efficient and transgene-free genome editing in wheat through transient expression of CRISPR/Cas9 DNA or RNA. Nat. Commun..

[29] Kim H (2017). CRISPR/Cpf1-mediated DNA-free plant genome editing. Nat. Commun..

[30] Davey MR, Anthony P, Power JB, Lowe KC (2005). Plant protoplasts: status and biotechnological perspectives. Biotechnol. Adv..

[31] Eeckhaut T, Lakshmanan PS, Deryckere D, Van Bockstaele E, Van Huylenbroeck J (2013). Progress in plant protoplast research. Planta.

[32] Gaj MD (2004). Factors influencing somatic embryogenesis induction and plant regeneration with particular reference to *Arabidopsis thaliana* (L.) Heynh. Plant Growth Regul..

[33] Carimi F, De Pasquale F, Crescimanno FG (1999). Somatic embryogenesis and plant regeneration from pistil thin cell layers of citrus. Plant Cell Rep..

[34] Paul H, Belaizi M, Sangwan-Norreel BS (1994). Somatic embryogenesis in apple. J. Plant Physiol..

[35] Fischer R, Emans N (2000). Molecular farming of pharmaceutical proteins. Transgenic Res..

[36] Pogue GP (2010). Production of pharmaceutical‐grade recombinant aprotinin and a monoclonal antibody product using plant‐based transient expression systems. Plant Biotechnol. J..

[37] Krenek P (2015). Transient plant transformation mediated by *Agrobacterium tumefaciens*: principles, methods and applications. Biotechnol. Adv..

[38] Wang M, Wang G, Ji J, Wang J (2009). The effect of *pds* gene silencing on chloroplast pigment composition, thylakoid membrane structure and photosynthesis efficiency in tobacco plants. Plant Sci..

[39] Xie K, Minkenberg B, Yang Y (2015). Boosting CRISPR/Cas9 multiplex editing capability with the endogenous tRNA-processing system. Proc. Natl Acad. Sci. USA.

[40] Andersson M (2017). Efficient targeted multiallelic mutagenesis in tetraploid potato (*Solanum tuberosum*) by transient CRISPR-Cas9 expression in protoplasts. Plant Cell Rep..

[41] Litz RE, Grosser JW (1998). Isolation, culture and regeneration of avocado (*Persea americana* Mill.) protoplasts. Plant Cell Rep..

[42] Jardak R., Mliki A., Ghorbel A. & Reustle G. L. Transfer expression of UIDA gene in grapevine protoplasts after PEG-mediated transformation. *Int. J. Vine Wine Sci*. (2002).

[43] Saito A, Suzuki M (1999). Plant regeneration from meristem-derived callus protoplasts of apple (*Malus domestica* cv.Fuji’). Plant Cell Rep..

[44] Maćkowska K, Jarosz A, Grzebelus E (2014). Plant regeneration from leaf-derived protoplasts within the *Daucus* genus: effect of different conditions in alginate embedding and phytosulfokine application. Plant Cell Tissue Organ Cult..

[45] Bonga J. M. *Cell and Tissue Culture in Forestry* (Springer, 1987)

[46] Peña L (2001). Constitutive expression of *Arabidopsis**LEAFY* or *APETALA1* genes in citrus reduces their generation time. Nat. Biotechnol..

[47] van Nocker S, Gardiner SE (2014). Breeding better cultivars, faster: applications of new technologies for the rapid deployment of superior horticultural tree crops. Hortic. Res..

[48] Wu H (2015). Genetic transformation of commercially important mature citrus scions. Crop Sci..

[49] Jacobs TB, Zhang N, Patel D, Martin GB (2017). Generation of a collection of mutant tomato lines using pooled CRISPR libraries. Plant Physiol..

[50] Iaffaldano B, Zhang Y, Cornish K (2016). CRISPR/Cas9 genome editing of rubber producing dandelion *Taraxacum kok-saghyz* using *Agrobacterium rhizogenes* without selection. Ind. Crops Prod..

[51] Altpeter F (2016). Advancing crop transformation in the era of genome editing. Plant Cell.

[52] Sood P, Bhattacharya A, Sood A (2011). Problems and possibilities of monocot transformation. Biol. Plant.

[53] Wilmink A, Dons JJ (1993). Selective agents and marker genes for use in transformation of monocotyledonous plants. Plant Mol. Biol. Rep..

[54] Gao J (2015). CRISPR/Cas9-mediated targeted mutagenesis in *Nicotiana tabacum*. Plant Mol. Biol..

[55] Li W (2016). An AGAMOUS intron‐driven cytotoxin leads to flowerless tobacco and produces no detrimental effects on vegetative growth of either tobacco or poplar. Plant Biotechnol. J..

[56] Li H, Durbin R (2009). Fast and accurate short read alignment with Burrows–Wheeler transform. Bioinformatics.

